# Feibi Recipe Reduced Pulmonary Fibrosis Induced by Bleomycin in Mice by Regulating BRP39/IL-17 and TGF*β*1/Smad3 Signal Pathways

**DOI:** 10.1155/2020/5814658

**Published:** 2020-10-12

**Authors:** Wei Wang, Zhaoheng Liu, Jie Niu, Haojie Yang, Qi Long, Haoge Liu, Xiaofeng Gu, Yang Jiao

**Affiliations:** ^1^Respiratory Department, Chongqing Traditional Chinese Medicine Hospital, No. 6 Pan Xi Qi Zhi, Jiangbei District, Chongqing 400021, China; ^2^School of Life Sciences, Beijing University of Chinese Medicine, Northeast Corner of the Intersection of Yang Guang Nan Da Jie and Bai Yang Dong Lu, Fangshan District, Beijing 102488, China; ^3^Dongfang Hospital Affiliated to Beijing University of Chinese Medicine, No. 6, Fang Zhuang, Fang Xing Yuan, Fengtai District, Beijing 100078, China; ^4^Beijing University of Chinese Medicine, No. 11 Bei San Huan Dong Lu, Chaoyang District, Beijing 100029, China

## Abstract

Fibrotic remodeling has become the result of many lung diseases, and these disorders can be categorized with known as well as unknown etiologies. Idiopathic pulmonary fibrosis is the most fatal disease among the unknown etiology. TGF*β*1/Smad3 signal pathway plays an important role in lung fibrosis and epithelial regeneration. This study investigated the effects and mechanism of Feibi Recipe (FBR) on pulmonary fibrosis. In this experiment, C57BL/6 mice were used and bleomycin was used to induce the lung injury. Meanwhile, the study showed a significant reduction in pathological response and mediators of inflammation and fibrosis such as IL-6, ICAM-1, IL-13, IL-17, BRP-39, TGF*β*1, Smad3, and Smad7 were identified. Collectively, the FBR appears to attenuate the lung injury and the modeling of fibrosis in mice.

## 1. Introduction

Idiopathic pulmonary fibrosis (IPF), regarded as a debilitating chronic lung disease [1], is characterized by progressive dyspnea, dry cough, shortness of breath, and wheezing with the average survival limited to 2-3 years, and the 5-year survival rate is less than 40% [2]. Lung transplantation is the only effective treatment option in eligible patients [1]. Until now, the mechanisms of the pathogenesis of this disease are not fully understood, and the main understanding of the pathogenesis includes inflammatory injury, oxidative stress, and epithelial-mesenchymal cells. Furthermore, there are more evidences indicating that the cause of the disease is associated with aberrant fibrotic tissue reparation [3].

Inflammatory factors such as interleukin-17 (IL-17), interleukin- 6 (IL-6), interleukin-13 (IL-13), and tumor necrosis factor-*α* (TNF-*α*) play an important role in the formation of alveolar epithelial cell (AEC) apoptosis and matrix fiber repair and are activated in the early stage of pulmonary fibrosis [4]. Breast regression protein 39 (BRP39)/chitinase-3-like protein 1 (YKL40), both in humans and mice, belongs to one of the eight proteins of the chitinase family, and the gene transcribing this protein belongs to an ancient gene family. YKL-40 can be expressed in a variety of human cells, including alveoli epithelial cells, macrophages, neutrophils, and fibroblasts [5]. Transforming growth factor-*β*1 (TGF*β*1) is regarded as one of the most potent profibrotic factors and plays a crucial role throughout the process of fibrotic formation [6] and the synthesis of ECM [7, 8]. Meanwhile, TGF*β*1 can affect Smads pathway: Zawel et al. [9] found that the Smad3 and Smad4 pathways of lung fibroblasts were activated after the stimulation of TGF*β*1, and this process triggered the transcription of TGF*β*1's target genes Collal and Timp-1, leading to increased expression of type I collagen.

FBR is a traditional Chinese medicine formula according to Professor Ping'an Zhou who has 50 years of clinical experience. Former research approved that FBR had the functions of inhibiting the phosphorylation of P38MAPK and promoting resolution of experimental lung tissue interacting with TGF*β*1 and IL-6. Similarly, FBR also reduces lung fibrosis by inhibiting fibroblast migration via the interaction of type III collagen [10]. These findings suggest that FBR has the potential to improve the development of pulmonary fibrosis and attenuate the fibrosis by its anti-inflammation function while inhibiting TGF*β*1 and Smad3 signaling.

## 2. Materials and Methods

### 2.1. Reagents and Materials

Mice IL-6 ELISA Kit (cat. number ab10072), Mice CXCL-13 ELISA Kit (cat. number ab212167), Mice ICAM-1 ELISA Kit (cat. number ab100688), IL-17 antibody (cat. number ab79056), TGF*β*1 antibody (cat. number ab64715), Smad3 antibody (cat. number ab40854), Smad7 antibody (cat. number ab216428), and CHI3L1 antibody (cat. number ab180569) were purchased from Abcam.

### 2.2. Preparation of Bleomycin

Bleomycin Sulfate for Injection (15U) was purchased from Fresenius Kabi, 0.9% Sodium Chloride Injection was purchased from Sichuan Kelun Pharmaceutical Co., Ltd. The concentration of bleomycin sodium chloride solution is 0.8 U/ml.

### 2.3. Animal Model and Experiment Design

44 C57BL/6 male mice were purchased from Beijing Vital River Laboratory Animal Technology Co., Ltd. After 7 days of adaptive feeding, the model was made. All mice were randomly assigned to sham group (8 mice), model group (12 mice), prednisone group (12 mice), and FBR group (12 mice). In all groups except the sham group, the lungs of mice were injured by bleomycin.

The mice were abdominally anesthetized by the mixture of ether and propylene with the scale of 2 : 3. 2 ml of the mixture was put into a plastic jar with several wholes on the lid, and then the mice were put into the jar. Winking reflex was used to test the depth of the anesthetization. The mice were vertically hung by the upper teeth. Then, their tongues were pulled out, and the bleomycin sodium chloride solution (0.8 U/ml) was injected into the mouth, the solution being evenly distributed in the lungs with the continuous coughing. From the 1st day, we gave saline (12.5 ml/kg·bw) to the model group by gavage once a day for 28 days. Meanwhile, the prednisone group was fed with prednisone sodium chloride solution (0.56 mg/kg·bw) and the FBR group with FBR (12.5 ml/kg·bw). The dosage of animals was converted according to the dosage of humans as the same frequency with the model group.

### 2.4. Deaths and Body Weight Changes Measurement

The deaths and the body weight changes were recorded on the 1st, 7th, 14th, 21st, and 28th day.

### 2.5. Pulmonary Function Test

Four of the mice in each group were anesthetized by intraperitoneal injection of 1% pentobarbital sodium. The neck skin and muscle were cut along the median line, the trachea was exposed and cut with a “T” shape incision, and a trachea cannula was inserted into the tube. Then, the mice were put into the body plethysmography and connected with the sensor of the pulmonary function instrument (AniRes 2005 animal pulmonary function system, Beijing Bestlab Science and Technology Ltd). The results were read using the computer.

### 2.6. Sample Collection

After the pulmonary function test, the mice were euthanized by exsanguination. The blood was drawn and centrifuged with 4°C, 3000 r/s, for 10 min. The supernatant was stored for test. After that, we replaced the trachea cannula by a trocar and drew out the metal needle fixing the plastic tube with the trachea by a suture. Bronchoalveolar lavage fluid (BALF) was collected by 0.5 ml 0.9% sodium chloride injection and suction, repeating 3 times. Other 4 of the mice in each group were directly anesthetized and euthanized by exsanguination without the pulmonary function test and the BALF collection. The blood was collected, and the lungs were separated into 2 parts. The left lobe lung was fixed by polyoxymethylene for HE, Masson, and immunohistochemical staining, while the right lobe lung was reserved in liquid nitrogen for Western Blot and RT-PCR.

### 2.7. Inflammation Cell Test in BALF

The BALF was tested by animal blood routine analyzer (HF-3800, Healife, China). The quantities of white blood cell (WBC), neutrophil (NEUT), and lymphocyte (LYMPH) were read using the analyzer.

### 2.8. General Histological Staining

The left lobe was fixed for more than 24 h, then dehydrated, embedded in paraffin, and cut into 5 *μ*m sections. The sections were (1) put into xylol I, II, each for 15 mins; (2) put into ethanol with concentrations of 100% I, II ⟶ 95% I, II ⟶ 90% ⟶ 80% ⟶ 70% ⟶ 60% ⟶ 50%, each for 2 min, and washed with running water for 5 min; (3) stained with hematoxylin for 15 min, with washing the extra hematoxylin; (4) put into 1% hydrochloric acid alcohol for 30 s and washed with running water for 10 min; (5) stained with eosin for 15 min and washed for 1 min; (6) put into ethanol with concentrations of 50% ⟶ 60% ⟶ 70% ⟶ 80% ⟶ 90% ⟶ 95% I, II ⟶ 100% I, II, each for 2 min; and (7) put into xylol I, II, each for 15 mins.

### 2.9. Masson Staining

The steps (3)–(5) were different from HE staining as follows (other phases were the same).

The sections were (1) stained with Bouin's solution for 12 h and washed with running water; (2) stained with Harris' hematoxylin for 15 min and washed with running water; (3) put into 1% hydrochloric acid alcohol for 30 s and washed with running water for 10 min; (4) stained with acid Ponceau for 10 min and washed by distilled water; (5) put into phosphomolybdic acid solution for 5 min; (6) stained with aniline blue for 5 min; and (7) put into glacial acetic acid for 1 min.

### 2.10. Immunohistochemical Staining

The sections were baked at 60°C for 30 minutes, microwave antigen retrieval was performed for 40 minutes, and then endogenous peroxidase was quenched with 3% H_2_O_2_ for 10 minutes at a room temperature and away from light. The sections were incubated with a primary antibody at 4°C for a night, and complete washing was performed by PBS, followed by secondary antibody for 20 minutes and washing again. The positive immunostaining in the tissues was visualized by 3,3-diaminobenzidine tetrahydrochloride (DAB) and then stained with hematoxylin. The positive expression of the cell was brown. The picture was observed by Olympus microscope (Tokyo, Japan). The data was analyzed by Image-Pro Plus 6.0 (Media Cybernetics, Rockville, MD, USA).

### 2.11. RT-PCR

The tissue was homogenized in liquid nitrogen. For quantitative RT-PCR, the primers were designed as follows: Actin, 5- GCCCTGAGGCTCTCTTCCA-3 (forward) and 5- GCGGATGTCGACGTCACA-3 (reverse); IL-17, 5- TCAGCGTGTCCAAACACTGAG-3 (forward) and 5- CGCCAAGGGAGTTAAAGACTT-3 (reverse); BRP39, 5- ATGCACACCTCTACTGAAGCC-3 (forward) and 5- ACCAGCTTGTACGCAGAGC -3 (reverse); TGF*β*1, 5- AGCTGCGCTTGCAGAGATTA-3 (forward) and 5- AGCCCTGTATTCCGTCTCCT-3 (reverse); Smad3 5- AGGGGCTCCCTCACGTTATC-3 (forward) and 5- CATGGCCCGTAATTCATGGTG-3 (reverse); Smad7, 5- GGGCTTTCAGATTCCCAACTT-3 (forward) and 5- AGGGCTCTTGGACACAGTAGA-3 (reverse). This was carried out according to the instructions of Trizol-Invitrogen. All assays were performed in triplicate and independently repeated three times. The gene expression level was normalized to the GAPDH, by the method of ΔΔCT, and relative gene expression data was calculated by 2^−ΔΔCT^ relative to the gene expression of mice in the control group.

### 2.12. Analysis of Protein in Lung Tissue Using Western Blot

Levels of IL-17, BRP39, TGF*β*1, Smad3, and Smad7 expression were analyzed. The tissue was homogenized in RIPA lysis buffer. The homogenate was centrifuged at 12000 r/min at 4°C (the centrifuge was obtained from Thermo, Legend Micro 21R) for 15 min, and the supernatant was collected. Protein concentration was determined with a BCA protein kit (MDL Biotech Co., Ltd.) and quantified to 0.5 mg/ml. The protein samples were separated on a 10% SDS polyacrylamide gel by SDS-PAGE electrophoresis system (Bio-Rad, USA) and then transferred to a polyvinylidene fluoride (PVDF) membrane (Millipore, USA). After that, the samples were blocked with 5% skimmed milk for 1 h and then incubated with primary antibodies (IL-17, BRP39, TGF*β*1, Smad3, and Smad7) at 4°C overnight. Then the samples were exposed to secondary antibody for 60 minutes at 37°C. ECL reagent was used to analyze the membranes. All assays were performed and repeated three times.

### 2.13. Cytokine Measurement Using ELISA

Levels of IL-6, ICAM-1, and CXCL-13 expression were measured with ELISA kits. All procedures were performed in accordance with the manufacturer's instructions.

### 2.14. Statistical Analysis

All graphing and statistical analyses were performed using SPSS 16.0. If the data met normal distribution, the results were shown as mean ± standard deviation (mean ± S. D); otherwise, the results were shown as median (interquartile range, *P*75–*P*25). Comparisons among multiple groups were analyzed with one-way ANOVA. Single comparisons were made with LSD test. If the data did not meet normal distribution, the results were analyzed with Kruskal–Wallis rank sum test. *P* values ≤ 0.05 were considered statistically significant.

## 3. Results

### 3.1. Deaths and Body Weight Changes

**Figure 1:**
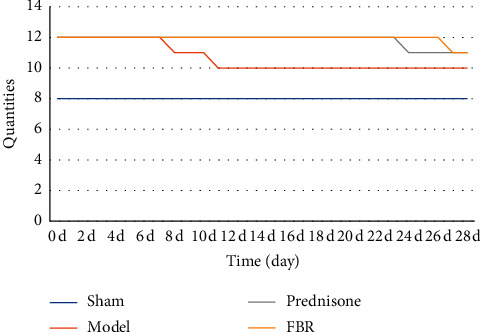
The mice in sham group did not die. Compared with mice in the model group, the number of deaths in prednisone and FBR group is less.

The mice in sham group did not die. However, in the model group, on the 7th and 10th day, 1 mouse died. In the prednisone group, on the 23rd and 26th day, 1 mouse died. In the FBR group, on the 26th day, 1 mouse died (Figure 1).

**Figure 2:**
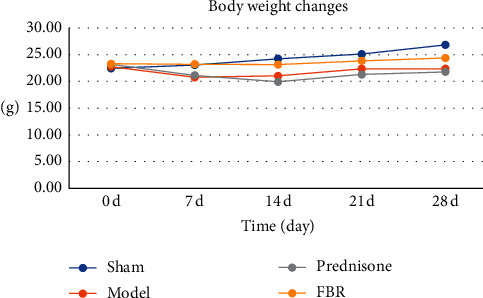
There was no statistically significant initial weight. On the 7th day and 14th day, statistical difference was found between sham group and model group (*P* < 0.05), sham group and prednisone group (*P* < 0.05), model group and FBR group (*P* < 0.05), and prednisone group and FBR group (*P* < 0.05). On the 21st day, statistical difference was found between sham group and model group (*P* < 0.05), sham group and prednisone group (*P* < 0.05), and prednisone group and FBR group (*P* < 0.05). On the 28th day, statistical difference was found between sham group and model group (*P* < 0.05), sham group and prednisone group (*P* < 0.05), model group and FBR group (*P* < 0.05), prednisone group and FBR group (*P* < 0.05).

The body weight of mice in the sham group increased steadily but decreased in the 1st week and increased in the following 3 weeks. The number in the prednisone and FBR group decreased in the 1st 2 weeks and then increased in the following 2 weeks (see Table 1 and Figure 2).

### 3.2. FBR Ameliorates Bleomycin-Induced Lung Injury

**Figure 3:**
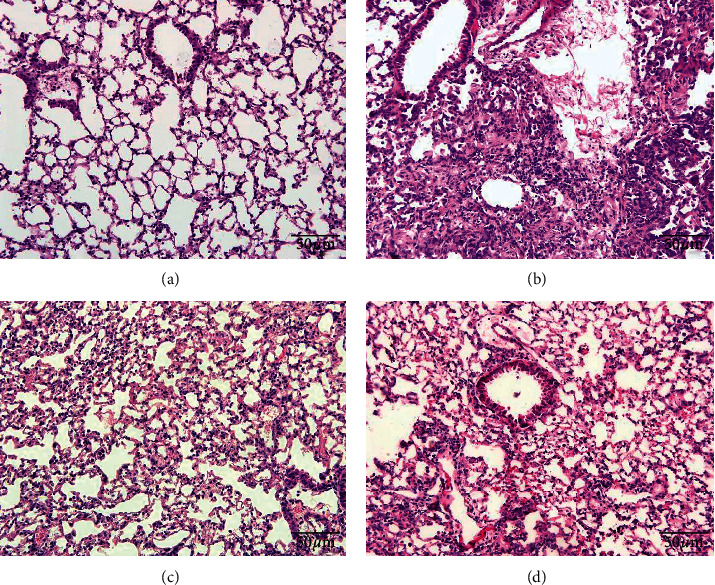
Effect of FBR on bleomycin-induced pulmonary histopathology in mice. There was less inflammation infiltration with FBR group and its positive control prednisone group. (a) Sham group (200x), (b) model group (200x), (c) prednisone group (200x), (d) FBR group (200x).

**Figure 4:**
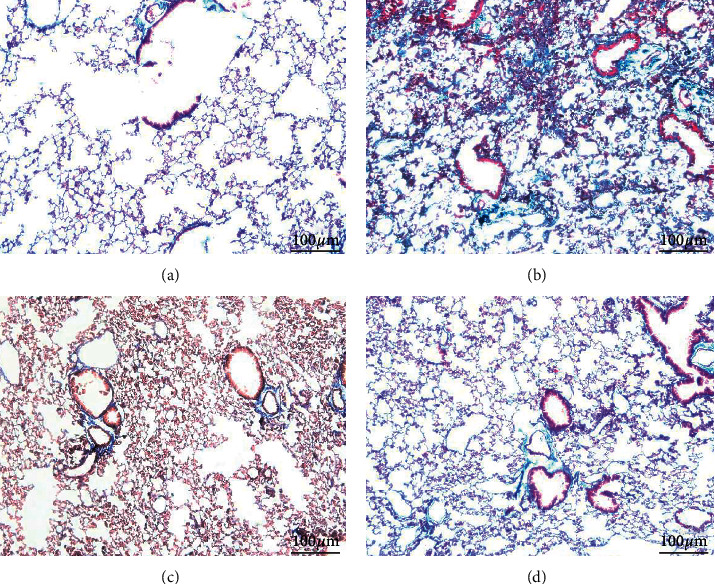
Effect of FBR on bleomycin-induced pulmonary fibrosis in mice. The fibrosis collagen deposition was improved in FBR group and its positive control prednisone group. (a) Sham group (100x), (b) model group (100x), (c) prednisone group (100x), (d) FBR group (100x).

HE and Masson staining were performed to evaluate pathological changes in lung tissues. In the model group, the inflammatory cells were infiltrated and the fibrous tissue was generated. Besides, alveolar collapse with consolidation and widened alveolar septum were shown. Compared with model group, FBR group showed that the inflammatory cell infiltration and the bronchial injury were improved, and the same improvements were seen in the prednisone group (Figures 3 and 4).

### 3.3. FBR Improves the Lung Function

The result showed that the FEV0.1, FEV0.2, and PEF were worse than those in the sham group (*P* < 0.05). The FBR and prednisone could obviously improve the FEV0.2 and PEF (*P* < 0.05) (Table 2).

### 3.4. FBR Inhibits Inflammation in Lungs

The WBC and neutrophil in the model group were increased compared with sham group (*P* < 0.05). Even though there is no statistical difference in these 2 indicators when compared with the other 2 groups (*P* > 0.05), still increasing trends were shown in the model group, which means that the FBR and prednisone had an anti-inflammatory function (Table 3).

### 3.5. Effect of FBR on Levels of IL-6, ICAM-1, and CXCL13 in Serum

The cytokines were measured by ELISA kits. All these 3 cytokines were greater in model group when compared with sham group (*P* < 0.05); FBR could significantly lower these 3 cytokines as the prednisone does (*P* < 0.05) (Table 4).

### 3.6. Immunohistochemical Staining

**Figure 5:**
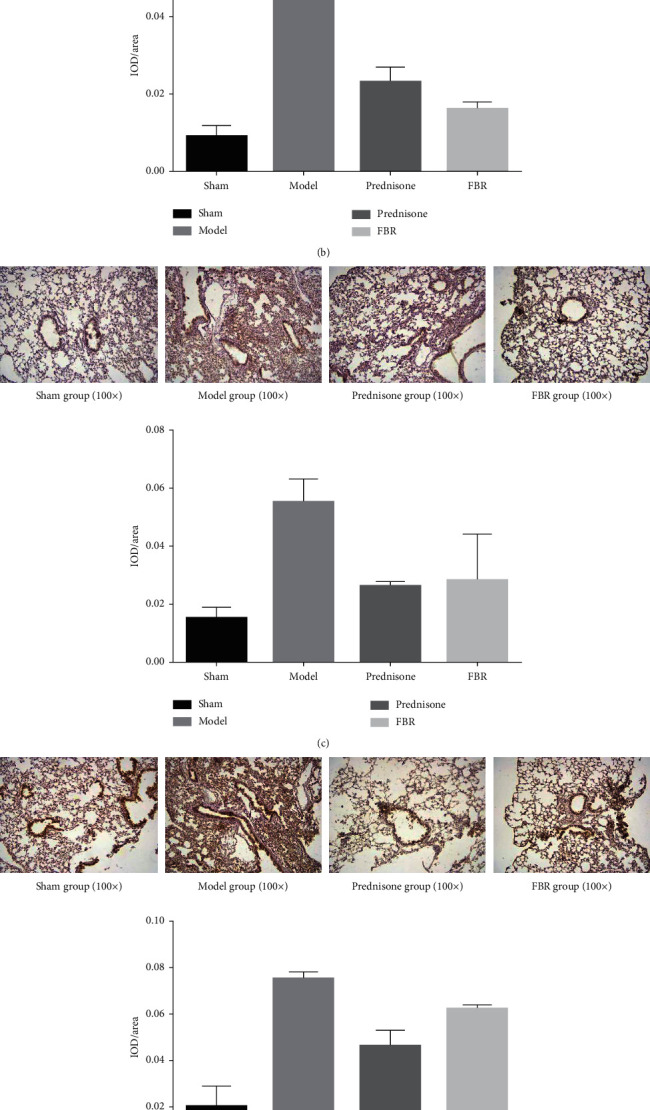
Comparison of the proteins IL-17 (a), BRP39 (b), TGF*β*1 (c), Smad3 (d), and Smad7 (e) in mice lung tissue with the method of immunohistochemical staining. The expression level of IL-17, BRP39, TGF*β*1, and Smad3 is significantly higher than that in sham group (*P* < 0.05). Compared with model group, the expression of BRP39, TGF*β*1, and Smad3 is lower in both FBR group and prednisone group (*P* < 0.05). There was no statistical difference between model group and FBR group in IL-17 (*P* > 0.05). The expression of Smad7 was relatively low in model group when compared with the other 3 groups (*P* < 0.05).

Immunohistochemical staining was used to evaluate the IL-17, BRP39, TGF*β*1, Smad3, and Smad7 proteins in lung tissues. Image-Pro Plus 6.0 was used to measure the parameter of IOD/area. From the analyzed results, we could find that the expression levels of IL-17, BRP39, TGF*β*1, and Smad3 were more significantly increased than those in the sham group (*P* < 0.05). FBR can inhibit the expression of BRP39, TGF*β*1, and Smad3 in lung tissues compared with model group (*P* < 0.05), and prednisone group also showed similar results. As for IL-17, there was no statistical difference between model group and FBR group, but the trend of increasing was shown in model group. The expression of Smad7 was decreased in model group when compared with the other 3 groups (*P* < 0.05) (Figure 5).

### 3.7. Effect of FBR on Levels of IL-17, BRP39, TGF*β*1, Smad3, and Smad7 Proteins in Lung Tissue

**Figure 6:**
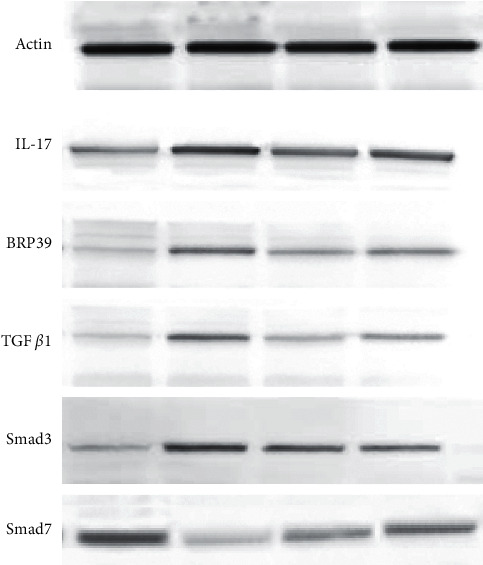
Comparison of the proteins IL-17, BRP39, TGF*β*1, Smad3, and Smad7 in mice lung tissue.

Western Blotting was used. Compared with the sham group, the IL-17, BRP39, TGF*β*1, and Smad3 proteins were upregulated and Smad7 was downregulated in the model group (*P* < 0.05); the FBR has the same effect of prednisone that could decrease the expression of BRP39 and TGF*β*1 (*P* < 0.05). There was no statistical significance when comparing IL17 and Smad3 in model group with FBR group (*P* > 0.05). In addition, FBR could significantly increase the expression of Smad7 (*P* < 0.05) (Figure 6 and Table 5).

### 3.8. Effect of FBR on Levels of the mRNA of IL-17, BRP39, TGF*β*1, Smad3, and Smad7 in Lung Tissue

The levels of IL-17, BRP39, TGF*β*1, and Smad3 in the model group were greater than these in sham group (*P* < 0.05), and FBR could lower the expression of BRP39, TGF*β*1, and Smad3 compared with these in model group (*P* < 0.05). There was statistical difference between the model group and FBR group when comparing the expression of Smad7, as FBR could inhibit the expression of IL-17 mRNA (*P* < 0.05) (Table 6).

## 4. Discussion

There is no effective treatment for IPF except lung transplantation which is expensive, and the source is hard to find. The drugs we have used before such as glucocorticoids, cyclophosphamide, cyclosporine A, and colchicine are not recommended for use since there is no evidence showing that they are effective. Pirfenidone and nidanib are currently recommended in the IPF guideline; however, the effectiveness and safety have not been clearly defined, and they have some side effects [11, 12]. Chinese medicine has the advantage of hitting multiple targets, but has less effect on the treatment of IPF, so we try to find medicines suitable for treating pulmonary fibrosis.

Bleomycin is a widely used medicine for treating tumors, and its main side effects is inducing lung fibrosis, so it is used as a medicine to induce lung fibrosis in mice models. From the result, we can find that it caused lung injuries such as inflammatory cell aggregation and pulmonary fibrosis. FBR could improve the lung injury as the positive control prednisone did. Meanwhile, there were also less inflammatory cells in the FBR group; we can know that FBR has an anti-inflammation function. Additionally, FBR also improved the survival rate and lung function after the modeling.

In this study, we detected levels of IL-6, ICAM-1, CXCL13 in serum and IL-17, BRP39, TGF*β*1, Smad3, and Smad7 in the lung tissue to explore the mechanism and signal pathways of lung injury induced by bleomycin and the effect of FBR at the gene and protein level.

IL-6 is a multifunctional cytokine and a key component of the inflammatory mediator network. As an anti-inflammatory cytokine or long-term cytokine, it can balance the damaging preinflammatory effects and play a protective role. Meanwhile, it has a two-way function of causing inflammation and anti-inflammation, which is related to the content in the tissue. Normal level is beneficial, and excessive secretion will cause inflammatory damage [13].

ICAM-1's receptor is LFA-1 and is expressed by neutrophils, eosinophils, and T lymphocytes. Meanwhile, the interaction between ICAM-1 and LFA-1 causes inflammatory cells to adhere to vascular endothelial cells and infiltrate into the bronchus. In addition, highly expressed ICAM-1 influences inflammatory cells adhesion to vascular endothelium and can mediate the endothelial transfer of inflammatory cells. The high expression of ICAM-1 helps to connect activated T cells and multinucleated leukocytes in the alveolar cavity and stimulate alveolitis [14, 15]. It also plays a synergistic role with TGF*β* and CTGF during the early stage of pulmonary fibrosis [16] and is involved in the pulmonary tissue inflammation and repair of pulmonary fibrosis.

CXCL-13 plays a very important role in the homing of B lymphocyte to inflammatory lesions. Studies have found that serum CXCL-13 levels in IPF patients are significantly higher than those in the control group and the COPD group. If IPF is associated with pulmonary hypertension or acute exacerbation, serum CXCL-13 will be much higher, which indicates that the situation can be fatal, or the patient needs urgent lung transplantation. Therefore, CXCL-13 in serum can be used as an early marker for the diagnosis of pulmonary fibrosis [17].

Wilson and his colleagues [18] found that bleomycin-induced lung fibrosis in mice depends on IL-17-related signaling pathways. IL-17 is involved in the development of inflammation. The IL-17 activates a series of downstream pathways such as mitogen-activated protein kinases (MAPK) and nuclear factor kB (NF-kB) through synergy and negative regulation to affect the expression of IL-6, IL-8, and CXC chemokine ligand-5 (CXC5) which play important roles in inflammatory diseases. At the same time, IL-17 has a synergistic effect on TNF-*α*-induced IL-6.

Excessive increase of BRP39/YKL40 is related to various respiratory diseases such as inflammation, tissue repair, and airway remodeling [19]. BRP39/YKL40 also participates in the immune inflammatory response of pulmonary fibrosis, promotes Th2 cell immunity, regulates the release of IL-13, and activates macrophages. When lung tissue is attacked by viruses, allergens, bleomycin, etc., AEC is damaged, which causes apoptosis of alveolar type II epithelial cells, and then the alveolar macrophages will be activated, which increases BRP39/YKL40 expression and activates T lymphocytes to become Th2 and Th17 cells to release inflammatory factors such as IL-6 and IL-17, which promtes the fibroblasts to release TGFβ1 and introduce signals into cells and mediates the proliferation of myofibroblasts through Smad3, MAPK, c-Jun N-terminal kinase(JNK), and P38 MAPK pathway. In this way, the process of tissue damage and repair is repeated, promoting the excessive deposition of collagen and fibrosis formation. [20].

To summarize, the inflammation is regarded as the initial phase of pulmonary fibrosis that activates the next phase in which TGF*β*1 is secreted. From the result, we can know that FBR lowers the expression of inflammation in serum, for example, IL-6, ICAM-1, and CXCL13, and also inhibits the expression of IL-17 and BRP39 genes and the transcription of IL-17 protein.

TGF*β*1 is very essential in tissue growth and repair and is secreted by alveolar type II epithelial cells, alveolar macrophages, etc. Furthermore, it is one of the most powerful profibrotic factors [6].

TGF*β*1 promotes the synthesis of ECM in multiple stages [7, 8]. The occurrence of IPF is the result of the imbalance between the synthesis and degradation of ECM. TGF*β*1 is the strongest deposition promoter that enables ECM synthesis increase and degradation decrease as follows: (1) It upregulates the transcription and translation of matrix component genes. For example, it promotes the increase of mRNA expression levels of collagen and fibronectin as well as formation of extracellular matrix receptors, thus promoting the deposition of ECM. (2) It selectively inhibits collagenase and elastase synthesis and activation to accumulate ECM in the lungs and thicken the alveolar wall. TGF*β*1 also promotes the transformation from fibroblast to myofibroblast.

Smad pathway can be affected by TGF*β*1. Flanders [21] found that the TGF*β* profibrotic activities relied on the Smad3 signaling pathway. Chemotaxis of TGF*β* does not show response in the cells which do not contain Smad3. Mice without Smad3 gene are resistant to radiation-induced skin fibrosis, bleomycin-induced lung fibrosis, and carbon tetrachloride-induced liver fibrosis.

Smad7 is a negative feedback factor for TGF*β*. Its main mechanism of action is to recruit TGF*β* receptors through the E3 linker Smurf1/2 or Nedd4-2, leading to ubiquitination of the receptors. In bronchial epithelial cells, Smad7 can be induced by INF-*γ* expression to inhibit the TGF*β* signaling pathway [22]. Smad7 can also bind to the TGF*β*1 receptor and compete with the Smad3 protein for binding sites. In this way, it can inhibit Smad3 activation to inhibit TGF*β* [23].

Therefore, FBR may control its protein synthesis by downregulating the gene expression of TGF*β*1 and Smad3 in the lung tissue and upregulating the gene expression and protein synthesis of Smad7 to promote its negative feedback regulation of the TGF*β* to reduce bleomycin-induced pulmonary fibrosis in mice.

In addition, prednisone was used as a positive control drug in this experiment, and it was found that it can inhibit the expression of collagen I and collagen II of bleomycin-induced pulmonary fibrosis to inhibit the secretion of collagen fibers; inhibit the expression of inflammatory factors such as IL-6 and profibrotic factors such as TGF*β*1; and increase the content of antioxidants such as HYP to play an antifibrotic role. In this way the FBR compared with the prednisone can demonstrate effectiveness.

A former study proved that Feibi Recipe can effectively inhibit the overexpression of IL-6/TGF*β*1/P38MAPK cell signaling pathway and reduce pulmonary fibrosis of the bleomycin model rat. Therefore, to deduce the biological mechanisms of Feibi Recipe, suppressing immunopathological damage of pulmonary fibrosis may function by regulating the interaction of immune inflammatory and fibrosis factor. It was found that chitinase is the first cytokine released after macrophage activation, as well as the upstream factor of pulmonary fibrosis cytokine network, which participates in immune inflammatory pathological damage of pulmonary fibrosis. On the basis of the previous project, this study focused on gene and protein level, experimenting on bleomycin-induced model mice, aiming to study the impact of Feibi Recipe on the signaling pathway and interaction of immune inflammatory and fibrosis factor, and target of its intervention in immunopathological injury of pulmonary fibrosis. The research discussed the molecular mechanisms of Feibi Recipe in suppressing the immunopathological damage of pulmonary fibrosis, intended to provide evidence to enrich the treatment for pulmonary fibrosis, and built a foundation in the study of biological mechanism of interaction of immune inflammatory and fibrosis factor in immunopathological injury of pulmonary fibrosis.

## 5. Conclusions

We noted that FBR could reduce the lung injury induced by bleomycin, including stimulated inflammatory factors such as IL-6, ICAM-1, and CXCL-13 in serum, by regulating BRP39/IL-17 and TGF*β*1/Smad3 pathway. Meanwhile, the FBR could improve the lung function of the pulmonary fibrosis mice model.

## Figures and Tables

**Table 1:** Comparison of body weight changes in the 4 groups.

Group	0 d	7 d	14 d	21 d	28 d
Sham	22.45 ± 0.95	23.08 ± 1.73	24.23 ± 2.14	25.11 ± 2.13	26.82 ± 0.98
Model	22.75 ± 1.21	20.78 ± 1.49*∗*	21.04 ± 1.80*∗*	22.35 ± 1.05*∗*	22.36 ± 1.29*∗*
Prednisone	23.19 ± 1.10	21.13 ± 2.01*∗*	19.94 ± 2.48*∗*	21.30 ± 2.61*∗*	21.78 ± 2.40*∗*
FBR	23.31 ± 1.09	23.21 ± 1.65Δ^	23.15 ± 1.48Δ^	23.84 ± 1.68^	24.39 ± 2.00*∗*Δ^

*∗*Compared with the sham group, *P* < 0.05; Δcompared with the model group, *P* < 0.05; ^compared with the prednisone group, *P* < 0.05.

**Table 2:** Comparison of FEV0.1, FEV0.2, and PEF in the 4 groups.

Group	FEV0.1 (ml)	FEV0.2 (ml)	PEF (ml/s)
Sham	0.525 ± 0.264	0.811 ± 0.120	7.275 ± 0.141
Model	0.485 ± 0.238*∗*	0.757 ± 0.289*∗*	6.956 ± 0.168*∗*
Prednisone	0.517 ± 0.251	0.802 ± 0.203Δ	7.150 ± 0.219
FBR	0.523 ± 0.250	0.817 ± 0.245Δ^	7.250 ± 0.195Δ

*∗*Compared with sham group, *P* < 0.05; Δcompared with the model group, *P* < 0.05; ^compared with the prednisone group, *P* < 0.05.

**Table 3:** Comparison of WBC, NEUT, and LYMPH in BALF in the 4 groups.

Group	WBC*∗*10^9^	NEUT*∗*10^9^	LYMPH*∗*10^9^
Sham	0.173 ± 0.050	0.083 ± 0.015	0.040 ± 0.030
Model	1.110 ± 0.195*∗*	0.560 ± 0.201*∗*	0.290 ± 0.347
Prednisone	0.390 ± 0.068	0.160 ± 0.052Δ	0.117 ± 0.086
FBR	0.490 ± 0.070	0.196 ± 0.650Δ	0.160 ± 0.100

*∗*Compared with the sham group, *P* < 0.05; Δcompared with the model group, *P* < 0.05.

**Table 4:** Comparison of cytokines in serum of mice in the 4 groups.

Group	IL-6	ICAM-1	CXCL-13
Sham	1.550 ± 0.080	43.43 ± 0.08	0.92 ± 0.40
Model	2.587 ± 0.215*∗*	76.2 ± 6.51*∗*	38.28 ± 5.20*∗*
Prednisone	1.972 ± 0.226*∗*Δ	45.12 ± 1.75Δ	21.34 ± 8.31*∗*Δ
FBR	2.027 ± 0.262*∗*Δ	51.55 ± 7.69Δ	22.04 ± 7.12*∗*Δ

*∗*Compared with the sham group, *P* < 0.05; Δcompared with the model group, *P* < 0.05.

**Table 5:** Comparison of the proteins IL-17, BRP39, TGF*β*1, Smad3, and Smad7 in mice lung tissue.

Group	IL-17	BRP39	TGF*β*1	Smad3	Smad7
Sham	0.28 ± 0.10	0.14 ± 0.05	0.13 ± 0.21	0.17 ± 0.06	0.57 ± 0.06
Model	0.65 ± 0.25*∗*	0.39 ± 0.07*∗*	0.36 ± 0.06*∗*	0.60 ± 0.07*∗*	0.13 ± 0.01*∗*
Prednisone	0.53 ± 0.02	0.29 ± 0.06*∗*	0.25 ± 0.08*∗*Δ	0.32 ± 0.10Δ	0.43 ± 0.20*∗*
FBR	0.45 ± 0.24	0.21 ± 0.05Δ	0.19 ± 0.22Δ	0.44 ± 0.15*∗*	0.32 ± 0.13*∗*Δ^

*∗*Compared with the sham group, *P* < 0.05; Δcompared with the model group, *P* < 0.05; ^compared with the prednisone group, *P* < 0.05.

**Table 6:** Comparison of the genes IL-17, BRP39, TGF*β*1, Smad3, and Smad7 in mice lung tissue.

Group	IL-17	BRP39	TGF*β*1	Smad3	Smad7
Sham	0.35 ± 0.17	0.38 ± 0.16	0.50 ± 0.04	0.58 ± 0.28	2.40 ± 0.97
Model	2.77 ± 0.21*∗*	4.27 ± 0.69*∗*	2.51 ± 0.38*∗*	2.91 ± 0.40*∗*	0.45 ± 0.12
Prednisone	0.94 ± 0.50*∗*	1.37 ± 0.03*∗*	1.13 ± 0.09*∗*Δ	1.13 ± 0.32	0.76 ± 0.20
FBR	0.93 ± 0.20*∗*	1.10 ± 0.27Δ	1.22 ± 0.08*∗*Δ	0.72 ± 0.14Δ	1.27 ± 0.09Δ

*∗*Compared with the sham group, *P* < 0.05; Δcompared with the model group, *P* < 0.05.

## Data Availability

The data used to support the findings of this study are available from the corresponding author upon request.
